# Direct Health Care Costs of Treating Seasonal Affective Disorder: A Comparison of Light Therapy and Fluoxetine

**DOI:** 10.1155/2012/628434

**Published:** 2012-10-18

**Authors:** Amy Cheung, Carolyn Dewa, Erin E. Michalak, Gina Browne, Anthony Levitt, Robert D. Levitan, Murray W. Enns, Rachel L. Morehouse, Raymond W. Lam

**Affiliations:** ^1^Sunnybrook Health Sciences Centre, 2075 Bayview Avenue, Toronto, ON, Canada M4N 3M5; ^2^Department of Psychiatry, University of Toronto, Toronto, ON, Canada; ^3^Centre for Addiction and Mental Health, Toronto, ON, Canada; ^4^Department of Psychiatry, University of British Columbia, Canada; ^5^School of Nursing, Ontario Training Centre in Health Services and Policy Research, McMaster University, Canada; ^6^Department of Psychiatry, University of Manitoba, Canada; ^7^Department of Psychiatry, Dalhousie University, Canada

## Abstract

*Objective.* To compare the direct mental health care costs between individuals with Seasonal Affective Disorder randomized to either fluoxetine or light therapy. *Methods.* Data from the CANSAD study was used. CANSAD was an 8-week multicentre double-blind study that randomized participants to receive either light therapy plus placebo capsules or placebo light therapy plus fluoxetine. Participants were aged 18–65 who met criteria for major depressive episodes with a seasonal (winter) pattern. Mental health care service use was collected for each subject for 4 weeks prior to the start of treatment and for 4 weeks prior to the end of treatment. All direct mental health care services costs were analysed, including inpatient and outpatient services, investigations, and medications. *Results.* The difference in mental health costs was significantly higher after treatment for the light therapy group compared to the medication group—a difference of $111.25 (*z* = −3.77, *P* = 0.000). However, when the amortized cost of the light box was taken into the account, the groups were switched with the fluoxetine group incurring greater direct care costs—a difference of $75.41 (*z* = −2.635, *P* = 0.008). *Conclusion.* The results suggest that individuals treated with medication had significantly less mental health care cost after-treatment compared to those treated with light therapy.

## 1. Introduction

Seasonal Affective Disorder (SAD) is a subtype of major depression that recurs on an annual basis. In the Diagnostic and Statistical Manual of Mental Disorders, Fourth Edition (DSM-IV), SAD is classified as a seasonal pattern specifier for major depressive disorder or bipolar disorder [1]. There is a significant burden of illness associated with SAD, and research demonstrates that individuals with SAD utilize significantly more health care services compared to age-matched controls without SAD [2–4]. 

There are two evidence-based treatments for SAD—antidepressants and light therapy. The efficacy of both of these treatments has been demonstrated in randomized controlled trials [5–7]. Studies have shown that individuals treated for SAD not only have improved symptoms but also have improved quality of life [8]. Light therapy and antidepressants are now recommended as first-line treatments for SAD in several clinical guidelines including the Canadian Network for Mood and Anxiety Treatments (CANMAT) [9], the American Psychiatric Association [10], the British Association of Psychopharmacology [11], and the World Federation of Societies of Biological Psychiatry [12]. 

The largest Canadian treatment study to date, CANSAD, compared antidepressants and light therapy in a randomized controlled trial and found that symptom reduction, response and remission rates, and improvement in quality of life were similar in patients with SAD treated with fluoxetine or light therapy [13]. However, there is little known about the direct mental health care costs for individuals treated for SAD and the cost differences between these two treatments. 

## 2. Aims of the Study

The objective of this study was to compare the direct mental health care costs between individuals with SAD randomized to either fluoxetine or light therapy using data from the CANSAD trial. 

## 3. Methods

### 3.1. Main Study and Findings

CANSAD was an 8-week multi-centre double-blind study that randomized participants to receive either active light therapy plus placebo capsules or placebo light therapy plus fluoxetine [13]. Briefly, the participants were aged 18–65 who met DSM-IV criteria for major depressive episodes with a seasonal (winter) pattern and were at least moderately depressed as determined by a baseline score of 23 or higher on the 24-Item Hamilton Depression Rating Scale (HAM-D). A total of 95 participants were included in this study sample (light therapy *n* = 48, fluoxetine *n* = 47). The active light therapy group had daily exposure to a white, 10,000 lux fluorescent light box for 30 minutes in the early morning. The active medication group received fluoxetine 20 mg once daily. There were 6 study visits: screening, baseline, weeks 1, 2, 4, and 8. Light therapy and fluoxetine both reduced SAD symptoms with no significant differences as measured by the HAM-D but those treated with fluoxetine endorsed more adverse effects such as palpitations. The two treatments had similar response and remission rates. The results also remained the same when the authors only included those individuals with more severe depression in the analysis. A full description of the study design and the main findings can be found elsewhere [8, 13]. 

### 3.2. Economic Evaluation

Mental health care service and drug use for CANSAD was collected for each subject for 4 weeks prior to the start of treatment and for 4 weeks prior to the end of treatment. Data were collected using the Health Utilization Study Instrument which has been used in previous research in Canada [14]. All direct mental health care services costs were analysed, including inpatient services, outpatient services, clinical and laboratory investigations, and medications. Direct care costs were divided into two categories: (1) mental health care costs and (2) prescription and over the counter drug costs. Mental health care costs were derived from the Approach to the Measurement of Costs Health Utilization Study [15]. Prescription drug costs were provided by the Drug Information Service, a regional independent drug information agency funded by the government based on drug costs for a large academic hospital in Southern Ontario, Canada. Prescription drug costs in Ontario are considered an average of costs among other jurisdictions in Canada. Over-the-counter drug costs were calculated using the average price of the drugs if they were purchased at (1) a discount pharmacy or (2) a nondiscount pharmacy. If an individual reported a generic name for a drug, the costs were determined on the generic version (see Table 1 for full list of services and drugs). All costs were calculated in Canadian dollars. Since both groups had the same number of visits throughout the course of the study, the costs of these visits were not included in our analyses.

The costs of the two study treatments were also included in the analysis. The cost of the fluoxetine was estimated at $40 for the course of the 8-week trial. The estimated upfront cost of the light box was $200 (total purchase price per unit). For comparison to the short-term costs of medication, we derived the cost of light therapy not based on the one-time up-front cost of the light box ($200) but rather the 2-month cost of a light box amortized over the lifetime use of the device. The amortized monthly cost of the device was calculated based on the 5 years of usual clinical use (and on 5 months of seasonal use per 12-month period) before the device or replacement bulbs are required for the device. This cost was estimated at $13.33 for the 8 weeks of this acute treatment trial. 

### 3.3. Analyses

The average direct mental health care cost differences before treatment (4 weeks prior to initiation of treatment) and after treatment (4 weeks prior to the end of treatment) for SAD for the two treatment groups were calculated. Since the data are not normally distributed, nonparametric tests (Wilcoxon rank-sum test) were conducted to examine for differences in average pre- and posttreatment costs between the two groups. To give a broader perspective on costs, we also examined the average cost differences between the two groups based on all direct health care costs (mental health and nonmental or general health related costs). 

We also conducted sensitivity analyses, to examine differences in costs using the full up-front purchase cost of the light box ($200) versus the amortized cost of $13.33 for the acute treatment phase. 

## 4. Results and Discussion

### 4.1. Results

The cost difference for mental health costs only was significantly higher after treatment for the light therapy group compared to the medication group—a difference of $111.25 (*z* = −3.77, *P* = 0.000) (see Table 2). Similarly, the cost difference in general health care costs (mental-health related and nonmental health or general health related costs) between the two treatment groups was also significantly higher in the light therapy group, although the difference was smaller at $29.12 and not statistically significant (*z* = −1.85, *P* = 0.64). 

We also conducted sensitivity analyses using the amortized cost of the light box. In contrast to the primary analyses, overall, the mental health care cost difference was significantly higher after treatment for the medication group compared to the light therapy group—a difference of $75.41 (*z* = −2.635, *P* = 0.008). The cost difference in general health care costs (mental-health related and non mental health or general health related costs) also remained higher for the medication group (*z* = −2.509, *P* = 0.012).

### 4.2. Discussion

This study examined the differences in direct mental health care costs of individuals with SAD treated within a randomized controlled trial comparing fluoxetine and light therapy treatment. Overall, the direct health care costs for patients with SAD were generally low with the average cost less then $200 over 4 weeks. This cost is less than half of the estimated direct care costs of individuals with stable major depression treated with antidepressants [16]. However, patients with SAD are often treated with combination treatment of both light therapy and antidepressants. The use of combination therapy could have a significant impact on the total direct care costs incurred by individuals with SAD.

The results suggest that in the acute 8-week treatment phase, participants treated with fluoxetine had significantly less mental health services cost compared to those treated with light therapy. However, the opposite results were found if the amortized cost of the light box was taken into account rather than the whole up-front cost of the purchase of a light box. Furthermore, although the light box is more expensive when initially purchased, it can be used during the entire episode with no extra cost and then again for the treatment of subsequent episodes over several years whereas the cost of medications is ongoing. This is particularly important in individuals with SAD because it is a recurrent illness and the depressive episodes tend to recur annually. For example, participants in the CANSAD study had experienced an average of 11 lifetime winter depressive episodes prior to study entry [13]. This is almost double the average number of episodes of participants with unipolar depression (average of 6 previous episodes) who were enrolled in the STAR*D treatment trial [17]. 

In addition, experts recommend that individuals with SAD initiate treatment several months prior to the start of winter each year in order to prevent onset of SAD and continue treatment until the end of winter, totaling an average of 5 months of treatment per calendar year [18]. Each $200 light box unit is expected to last for 5 years with low ongoing maintenance costs of approximately $25 over 5 years while the cost of fluoxetine is $24 per month. Therefore, the total cost of treating annual episodes of SAD for 5 years at 5 months of treatment per year would be $600 for fluoxetine and $200 for light therapy. Based on these estimates, light therapy is more expensive than fluoxetine in the first year of treatment but becomes cost saving after the second year of treatment (and for every subsequent year) compared to medications. In addition, the cost savings for light therapy would be even greater if (1) nongeneric medications, which have higher costs than the generic fluoxetine used in this study, were prescribed for treatment, or (2) the dose of fluoxetine was greater than 20 mg daily. 

Finally, in the sensitivity analyses where the amortized cost of the light box was used, those treated with fluoxetine also incurred greater costs when mental health and non-mental health care costs were both considered. One possible explanation for this finding is that individuals treated with fluoxetine had higher rates of some adverse effects such as gastrointestinal upset (e.g., nausea, diarrhea) and palpitations and therefore may utilize more health care services due to increased physical complaints compared to those treated with light therapy. 

A limitation to this study is that CANSAD was an acute treatment study and therefore, interpretation of the data is limited to the costs associated with short-term health services utilization of the CANSAD participants. In addition, costs associated with fluoxetine may or may not be similar to other antidepressants, particularly those where generics are still not available (e.g., duloxetine) and costs associated with the particular light box used in this study may or may not be generalizable to all light therapy units available on the market. However, the costs used in this study are representative of the average costs of generic antidepressant medications and light therapy units. In addition, patients were treated as part of a clinical trial, and health care costs in a “real world” situation may be different in unpredictable ways. However, this is the first study to report the direct care costs associated with treatment of SAD and therefore provides important information regarding the costs of managing this common and recurrent disorder. Patients often prefer nonmedication approaches in the treatment of depression [19], and this study suggests that the health care costs of light therapy may also be favourable compared to medication treatment, particularly in the long term. Given that there were no significant differences in response or remission rates found between the two treatments in the original study, health care providers such as government or private health insurance companies may be justified in simply choosing the most cost-saving treatment for individuals with SAD. 

## 5. Conclusions

This is the first study to compare the direct mental health care costs between individuals with SAD randomized to either medication or light therapy. The findings suggest that individuals treated with fluoxetine had significantly less mental health care cost after treatment compared to those treated with light therapy. However, if the cost of the light box is amortized, we find the opposite results—favouring light therapy. These results may be of value to patients and clinicians in considering the cost/benefit of SAD treatments and may assist health services payors with respect to support for these treatments.

## Significant Outcomes

1. Overall, direct care costs of individuals with Seasonal Affective Disorder were less expensive then the estimated care costs for those with unipolar depression.
2. With recurrent episodes of Seasonal Affective Disorder, light therapy is a cost-saving treatment option.
3. Using the amortized cost (over 5 years) instead of the upfront purchase cost of the light box in the analyses makes light therapy the cost-saving treatment option over fluoxetine for acute treatment.
4. If the up-front purchase cost of the light box (instead of the amortized cost over 5 years) is used in the analyses, fluoxetine is the cost-saving treatment over light therapy for acute treatment of SAD.

## Limitations

1. Data is limited to acute treatment.
2. Costing information is limited to direct care costs and excludes other societal costs.

## Figures and Tables

**Table 1:** Health care services and classes of drugs used by study participants.

Type of health care services	Classes of drugs
Emergency services	Analgesics
Inpatient services	Anxiolytics
Laboratory services	Antibiotics/antimicrobials
Medical imaging	Antidepressants
Nursing	Antihypertensives
Occupational therapy	Antilipids
Physician services	Birth control
Physiotherapy	Decongestants
Psychologist services	Hormone replacement therapy
Social work	Nutritional supplements/vitamins
	Sedatives
	Steroids

**Table 2:** Total health care costs and cost differences based on full purchase cost of light box between fluoxetine and light therapy arms.

Type of cost		Mental-health-related costs		Mental- and non-mental-health-related costs	
Treatment group		Medication	Light	Medication	Light
Direct health care costs	Before^1^	71.72 (158.68)	86.37 (203.68)	109.50 (186.77)	126.73 (238.13)
	After^2^	122.17 (117.75)	312.78 (186.09)	213.68 (263.29)	355.41 (234.47)

Drug costs	Before^3^	10.82 (21.74)	22.86 (56.93)	10.82 (21.74)	22.86 (56.93)
	After^4^	62.80 (70.60)	14.63 (55.46)	62.80 (70.60)	14.63 (55.46)

Total costs	Before^5^	82.35 (156.84)	109.23 (217.14)	120.18 (183.33)	149.60 (251.50)
	After^6^	184.95 (142.41)	327.41 (194.72)	276.48 (279.43)	369.77 (239.40)
	Mean of individual differences between before and after^7^	96.40 (206.29)*	207.65 (259.42)*	168.72 (302.09)**	197.84 (318.58)**

*P < 0.0001.

**Not statistically significant.

Notes: medication: fluoxetine 20 mg once daily, light therapy: light box 30 minutes per day, SD: standard deviation.

*Statistically significant differences were found between the fluoxetine group and light therapy group.

^†^Participants were randomized to either fluoxetine 20 mg once daily or light box 30 minutes each day.

^1^Total health care costs for the 4 weeks prior to enrollment in study.

^2^Total health care costs for the 4 weeks prior to the end of the study.

^3^Total drug costs for the 4 weeks prior to enrollment in study.

^4^Total drug costs for the 4 weeks prior to the end of the study.

^5^Total health care and drugs costs for the 4 weeks prior to enrollment in study.

^6^Total health care and drug costs for the 4 weeks prior to the end of the study.

^7^The difference in total health care and drug costs for the 4 weeks prior to the enrollment of the study and for the 4 weeks prior to the end of the study.

## References

[1] American Psychiatric Association (2000). *Diagnostic and Statistical Manual of Mental Disorders*.

[2] Eagles JM, Naji SA, Gray DA, Christie J, Beattie JAG (1998). Seasonal affective disorder among primary care consulters in January: Prevalence and month by month consultation patterns. *Journal of Affective Disorders*.

[3] Eagles JM, Howie FL, Cameron IM (2002). Use of health care services in seasonal affective disorder. *British Journal of Psychiatry*.

[4] Andrew JE, Wileman SM, Howie FL, Cameron IM, Naji SA, Eagles JM (2001). Comparison of consultation rates in primary care attenders with and without seasonal affective disorder. *Journal of Affective Disorders*.

[5] Lam RW, Gorman CP, Michalon M (1995). Multicenter, placebo-controlled study of fluoxetine in seasonal affective disorder. *American Journal of Psychiatry*.

[6] Terman M, Terman JS, Ross DC (1998). A controlled trial of timed bright light and negative air ionization for treatment of winter depression. *Archives of General Psychiatry*.

[7] Eastman CI, Young MA, Fogg LF, Liu L, Meaden PM (1998). Bright light treatment of winter depression: a placebo-controlled trial. *Archives of General Psychiatry*.

[8] Michalak EE, Murray G, Levitt AJ (2007). Quality of life as an outcome indicator in patients with seasonal affective disorder: results from the Can-SAD study. *Psychological Medicine*.

[9] Ravindran AV, Lam RW, Filteau MJ (2009). Canadian Network for Mood and Anxiety Treatments (CANMAT) Clinical guidelines for the management of major depressive disorder in adults. V. Complementary and alternative medicine treatments. *Journal of Affective Disorders*.

[10] American Psychiatric Association (2000). Practice guideline for the treatment of patients with major depressive disorder. *The American Journal of Psychiatry*.

[11] Anderson IM, Ferrier IN, Baldwin RC (2008). Evidence-based guidelines for treating depressive disorders with antidepressants: a revision of the 2000 British Association for Psychopharmacology guidelines. *Journal of Psychopharmacology*.

[12] Bauer M, Whybrow PC, Angst J, Versiani M, Möller HJ (2002). World Federation of Societies of Biological Psychiatry (WFSBP) Guidelines for Biological Treatment of Unipolar Depressive Disorders, Part 1: acute and continuation treatment of major depressive disorder. *The World Journal of Biological Psychiatry*.

[13] Lam RW, Levitt AJ, Levitan RD (2006). The can-SAD study: a randomized controlled trial of the effectiveness of light therapy and fluoxetine in patients with winter seasonal affective disorder. *American Journal of Psychiatry*.

[14] Browne G, Gafni A, Roberts J (2006). The health and social service utilization survey. *System-Linked Research Unit*.

[15] Browne G, Gafni A, Roberts J (2006). Approach to the measurement of costs (Expenditures) when evaluating health and social programmes. *System-Linked Research Unit*.

[16] Birnbaum HG, Ben-Hamadi R, Greenberg PE, Hsieh M, Tang J, Reygrobellet C (2009). Determinants of direct cost differences among US employees with major depressive disorders using antidepressants. *PharmacoEconomics*.

[17] Trivedi MH, Rush AJ, Wisniewski SR (2006). Evaluation of outcomes with citalopram for depression using measurement-based care in STAR*D: Implications for clinical practice. *American Journal of Psychiatry*.

[18] Westrin A, Lam RW (2007). Long-term and preventative treatment for seasonal affective disorder. *CNS Drugs*.

[19] Van Schaik DJF, Klijn AFJ, Van Hout HPJ (2004). Patients’ preferences in the treatment of depressive disorder in primary care. *General Hospital Psychiatry*.

